# Flow-Data Gathering Using NetFlow Sensors for Fitting Malicious-Traffic Detection Models

**DOI:** 10.3390/s20247294

**Published:** 2020-12-18

**Authors:** Adrián Campazas-Vega, Ignacio Samuel Crespo-Martínez, Ángel Manuel Guerrero-Higueras, Camino Fernández-Llamas

**Affiliations:** Department of Mechanical, Computer Science and Aerospace Engineering, Campus de Vegazana s/n, University of León, 24071 León, Spain; icrem@unileon.es (I.S.C.-M.); am.guerrero@unileon.es (Á.M.G.-H.); camino.fernandez@unileon.es (C.F.-L.)

**Keywords:** NetFlow, packet flow, advanced persistent threat, malicious traffic, dataset

## Abstract

Advanced persistent threats (APTs) are a growing concern in cybersecurity. Many companies and governments have reported incidents related to these threats. Throughout the life cycle of an APT, one of the most commonly used techniques for gaining access is network attacks. Tools based on machine learning are effective in detecting these attacks. However, researchers usually have problems with finding suitable datasets for fitting their models. The problem is even harder when flow data are required. In this paper, we describe a framework to gather flow datasets using a NetFlow sensor. We also present the Docker-based framework for gathering netflow data (DOROTHEA), a Docker-based solution implementing the above framework. This tool aims to easily generate taggable network traffic to build suitable datasets for fitting classification models. In order to demonstrate that datasets gathered with DOROTHEA can be used for fitting classification models for malicious-traffic detection, several models were built using the model evaluator (MoEv), a general-purpose tool for training machine-learning algorithms. After carrying out the experiments, four models obtained detection rates higher than 93%, thus demonstrating the validity of the datasets gathered with the tool.

## 1. Introduction

Advanced persistent threats (APTs) are one of the most concerning threats that companies, research centers, and governments have to face. APTs are defined in [1] as cyberattacks executed by sophisticated and well-resourced adversaries targeting specific information in high-profile companies and governments, usually in a long-term campaign involving different steps. Such threats use a large number of techniques to achieve their goals, such as port scanning, malware distribution, lateral network movements, privilege escalation, zero-day attacks, and social engineering. The use of a large number of techniques and the complexity of the attacks makes detecting APTs a complex problem for which no solution has been found yet.

One of the features that distinguish an APT is the malicious-traffic generation, both in phases of recognition and data exfiltration. In the literature, we can find numerous works that used artificial intelligence to detect malicious traffic. Specifically, the most common approach to detect such traffic is using supervised learning models. For instance, in [2], the authors proposed a network-anomaly detection system using classification algorithms trained with the UNSW-NB15 dataset. The authors obtained a 94.36% accuracy score using an Averaged one-dependence estimator (AODE), a 92.70% accuracy score using a Bayesian network, and a 75.73% accuracy score using naive Bayes. In [3], the authors carried out similar work. They used the same dataset for obtaining an efficient detection model with a high detection rate and low run time. Results provided by the authors were similar to those provided in [2], concluding AODE was the best algorithm with a 97.26% accuracy score and an approximate running time of 7 s. Research carried out in [4] proposed a hybrid model using bagging and rotation-forest techniques. To test the models, the authors used the NSL-KDD and UNSW-NB15 datasets obtaining an 85.8% accuracy score. Using a hybrid method, Reference [5] proposed a graphical-feature-based approach obtaining a 98.54% accuracy score with a model using a K-nearest neighbors (KNN) algorithm. In [6], the authors carried out a literature review map in order to establish the most commonly used datasets and algorithms to detect cyberattacks. Results showed that the UNSW-NB15 dataset provided good performance when classifying network traffic. Furthermore, the authors concluded that the algorithms with higher accuracy scores according to the literature are KNN, decision tree, and Naive Bayes (NB).

In the above-mentioned works, network-traffic datasets are gathered using the packet capture (pcap) API. Pcap files contain all network packets traveling over a computer network, including their payload. Models fitted with such datasets, despite their high performance, are not suitable for routers that manage a high rate of traffic per second. In such cases, due to performance constraints, it is impossible to analyze all network traffic data. Thus, in order to analyze network traffic in such environments, router manufacturers use packet-flow technologies.

A flow is defined as a set of IP packets passing an observation point in the network during a certain time interval. All packets belonging to a particular flow have a set of common properties such as source and destination IP addresses, and source and destination port numbers [7,8]. Some features that flow data gather are the number of packets in the flow, IP protocol type, protocol flags, and timestamp. The most used packet-flow technology is NetFlow, which is a network protocol designed by Cisco that aims to gather IP flow statistics [9]. NetFlow is the de facto standard to gather flow data. It is deployed in most commercial routers. For instance, it is used to monitor the network traffic of educational and research centers throughout Spain, and it is managed through the Spanish Network for Interconnection of Computer Resources (RedIRIS) [10].

On the other hand, synthetic-traffic generation is not a simple issue. Throughout the literature, we can find several tools to simulate network traffic with different purposes. In [11], the authors presented an IP traffic generator based on a specific hardware architecture that generates network traffic to perform tests in the development process of network elements. A comparative study of various traffic-generator tools was performed in [12], comparing the performance of several network-evaluation tools, namely, PackETH, Ostinato, and D-ITG. The performance of such tools was evaluated in terms of bandwidth utilization. A fast, scalable, and programmable traffic generator for the performance evaluation of high-speed networks is shown in [13]. The authors designed a hardware board to generate large amounts of randomized network traffic. Harpoon is a tool that generates TCP and UDP packets between hosts [14]. It is based on seven features of flow-based traffic. Harpoon tests the responsiveness of routers and networks. Besides difficulties in generating network traffic, using tools such as those described in the above works is not easy. To make it easier, a wrapper for traffic generators was proposed in [15].

As mentioned above, researchers focus on generating network traffic to test infrastructures. Therefore, these tools are not suitable to detect the malicious traffic generated by APTs in some phases. Consequently, in this work, we define a framework to gather flow datasets using the NetFlow specification. The framework allows for easily generating taggable network traffic to build datasets suitable for fitting classification models. We also present DOROTHEAs, a Docker-based solution that implements the above framework. It is highly scalable since users can easily incorporate their own customized scripts for both malicious and benign traffic. Users can also define complex virtual computer networks.

The remainder of the paper is organized as follows. Section 2 describes the materials and tools used in the work and the used method to evaluate DOROTHEA, which includes the generation of a dataset later used to fit a classification model than allows for port scanning attack detection. Section 4 shows the obtained results in the above experiments. Results are discussed in Section 5. Lastly, conclusions are presented in Section 6.

## 2. Materials and Methods

This section presents the materials, carried-out experiments, and the methods that evaluated them. First, we propose the guidelines to gather suitable flow datasets for fitting malicious-traffic detection models. Next, we present DOROTHEA, a Docker-based solution that follows the above guidelines.

### 2.1. Flow-Data Generation

In order to analyze network traffic in such environments, router manufacturers use packet-flow technologies. NetFlow is the de facto standard to gather flow data. NetFlow was originally developed by Cisco and later standardized by the IETF from version 9 of the protocol [9]. NetFlow services provide IP flow statistics. A flow is defined as a unidirectional sequence of packets with some common properties that pass through a network device [16]. Network elements, such as routers and switches, gather flow data and export them to collectors. Collected data provide fine-grained metering for highly flexible and detailed resource-usage accounting. Since NetFlow discards packet payloads, it is a very light protocol.

As mentioned in Section 1, network-traffic generation is not an easy-to-solve issue, especially when tagged data of both benign and malicious traffic are required for fitting malicious-traffic detection models. Most examples in the literature propose ad hoc solutions for specific problems and they are not suitable for different situations. Moreover, ad hoc solutions are not usually reproducible or scalable. A general-purpose method for gathering tagged flow data is required. This work proposes a framework for building flow-based network-traffic datasets.

**Figure 1:**
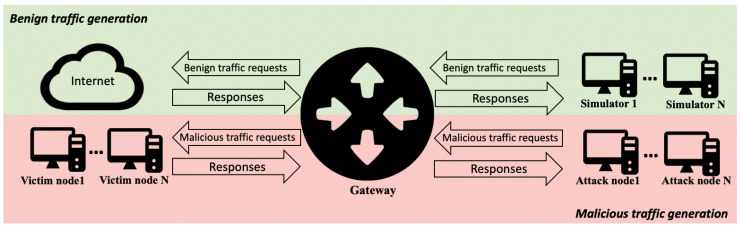
Flow-data generation framework.

Our proposal, as shown in Figure 1, has two main components. First, a legitimate-traffic generation method is required. We cannot use network traffic gathered from legitimate users since we cannot empirically assert all network traffic to be benign. In addition, in order to protect user confidentiality, using their data may not be allowed according to most data-protection regulations [17]. Thus, we propose to use network-traffic simulators so that we can empirically assert that the flow data gathered are benign without compromising user confidentiality. These simulators must generate the network traffic of the most commonly used protocols such as HTTP/HTTPS, SMTP, and SSH. A method to customize and add new network-traffic simulators is required in order to ensure that the framework could be used in different situations.

On the other hand, we need malicious traffic for fitting detection models. It is even more difficult to empirically assert that such traffic is malicious. Therefore, a malicious-traffic generation method is required. We propose to simulate cyberattacks using the most commonly used methods and techniques: port scanning, brute-force, dictionary attacks, injections, and denial of service. With regard to benign-traffic generation, an easy method is required to customize cyberattacks and to add new ones.

In both legitimate- and malicious-traffic generation, scalability is essential. We require a method that allows us to simulate realistic and complex network topologies that include legitimate, attack, and victim nodes.

Lastly, once traffic generation and network topology are defined, we need a methodology to gather flow data. We propose to use a NetFlow sensor installed in a gateway that is in charge of routing network traffic, and building and storing flow data. Regarding flow-data collection, to simulate realistic environments, we must define a threshold for packet gathering. To prevent congestion, commercial routers apply such thresholds to avoid the processing of all packets when building flow data. This means that not all packets are processed in order to build flow data. For instance, at Castilla y León, the entity in charge of routing all network traffic corresponding to RedIRIS is RedCAYLE, which is managed by Supercomputación Castilla y León (SCAYLE). Network-traffic management is conducted through two main routers located at León, and a series of switches and auxiliary routers distributed throughout the region. Even when using NetFlow Version 5, the data load is too big to analyze all packets routed through RedCAYLE’s routers. To prevent congestion, routers use a sample of all packets to build flow data. The rate of packets that RedCAYLE’s routers process is 1 out of 1000.

### 2.2. DOROTHEA

Next, we present DOROTHEA, a Docker-based solution that, according to the above proposals, allows for us to build packet-flow datasets (DOROTHEA’s technical report is available online, doi:10.5281/zenodo.4114119). We decided to use Docker due to its flexibility and scalability [18,19]. It allows for creating complex virtual computer networks deploying a large number of computers running different services, thereby simulating a realistic environment.

DOROTHEA uses a NetFlow sensor that collects the streams of packets that pass through a network interface. It allows for simulating a complex and realistic computer network. It is easily customizable and scalable. It is possible to set the number of computers that generate network traffic in a complex network topology. A threshold can be set to establish the number of used packets to create flows, thereby emulating the real functionality of routers. DOROTHEA allowed us to simulate both benign and malicious network traffic.

#### 2.2.1. Benign-Traffic Generation

**Figure 2:**
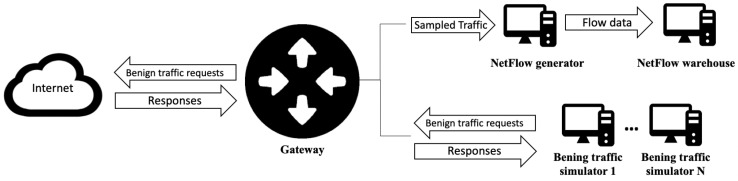
Benign-traffic generation scheme.

Benign traffic is network traffic that a real user generates when using a web browser, a mail client, or a remote desktop client, or when performing any other legitimate task. To obtain benign traffic, DOROTHEA uses network-traffic simulators that send packages to a gateway. Simulators use scripts for legitimate-traffic generation. These scripts are isolated items. Users may customize them or even incorporate their own. Network traffic generated by simulators is received by the gateway that performs two main tasks replicating commercial-router behavior. On the one hand, it routes packets to the Internet. On the other hand, it sends 1 out if X to a flow-data generator. X is the sampling threshold set by the user. As mentioned above, packet sampling is used in commercial routers in order to decrease the number of packets to be processed in order to generate flow data and reduce router congestion. Figure 2 shows the proposed architecture to generate benign traffic.

Regarding gateway behavior, the sampling threshold is relevant. Flow built without a sampling threshold to process packets contains data from a greater number of packets and therefore more information than that of flow built without considering all packets that pass through the network interface. This has a huge impact on a dataset, and a consequently negative impact on classification models fitted from such a dataset.

The sampling threshold applied to the sent packets is also applied to their associated reply packets from the Internet. The selected packets are sent to the flow-data generator, which has an *ipt_netflow* sensor installed (source code is available online on https://github.com/aabc/ipt-netflow). The sensor processes the network traffic and provides flow data in NetFlow V5-, V9-, and IPF formats. Flow data are sent to a NetFlow warehouse every 2 min.

#### 2.2.2. Malicious-Traffic Generation

**Figure 3:**
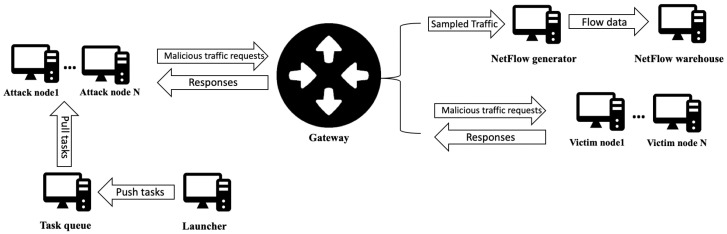
Malicious-trafficgeneration scheme.

Regarding malicious-traffic generation, Figure 3 shows the proposed architecture. Attacks are distributed between different nodes. In order to do so, DOROTHEA uses Celery, an open-source Python library that uses an asynchronous task queue to distribute jobs between different nodes. The user defines the number of nodes that are to carry out the attack, and the number of nodes that are to be attacked. Attack nodes use scripts to perform attacks. Such scripts are isolated items, so users may customize or even incorporate their own.

Once malicious-traffic generation is running, the launcher node loads attack scripts and enters the tasks into the queue. From the task queue, attack nodes obtain their tasks and start running them.

Attack nodes are connected to a gateway. The gateway, NetFlow generator, and NetFlow warehouse work as explained above for benign-traffic generation. Once all attack scripts are run, and all tasks in the queue are completed, DOROTHEA saves flow data and shuts all nodes down.

An essential DOROTHEA feature is that malicious-traffic generation is isolated from the Internet. Therefore, we ensure that all network traffic used to build flow data corresponds to malicious attacks so as to facilitate data labeling.

## 3. Evaluation

To evaluate the proposed framework, we used a dataset gathered by using DOROTHEA to fit classification models that could detect port-scanning attacks. The next section provides an in-depth explanation about data gathering, and the methodology used to design and evaluate the classification models.

### 3.1. Data Gathering

We gathered a dataset for fitting classification models using DOROTHEA. Specifically, we built models to detect port-scanning attacks. In order to ensure model generalization, two different datasets were gathered. The first was used to train models, while the second was used to test them. Using a different dataset allowed for us to assert that our classification models could maintain their detection rate up in different network environments.

Both the training dataset ($D_{1}$) (available online, doi:(10.5281/zenodo.4106730) and the test dataset ($D_{2}$) (available online, doi:10.5281/zenodo.4106738) contained approximately 50% benign flow data and 50% malicious flow data. Benign flow data, corresponding to legitimate network traffic, were labeled “0”. Malicious flow data, corresponding to port-scanning attacks, were labeled “1”. Both datasets are freely available online under an open-access license.

Benign flow data at both datasets were generated using three Python scripts. The scripts are available online with an open-source license (https://niebla.unileon.es/cybersecurity/dorothea/-/tree/master/labs/lab_normal/generator/generate-traffic). The first simulates a user browsing the Internet using different search engines, thus generating HTTP and HTTPS network traffic. The second script simulates email sending using an SMTP protocol. The third script simulates SSH connections.

Malicious flow data at both datasets were generated using Nmap. Different types of port scanning on both TCP and UDP ports were carried out: TCP SYN; TCP Connect; UDP; TCP NULL, FIN, and Xmas; TCP ACK; TCP Window; and TCP Maimon scanning [20]. Port-scanning attacks were carried out by 100 attack nodes that scanned 65,536 ports on 200 victim nodes. The Python scripts used to perform the attacks are also available online with an open-source license (https://niebla.unileon.es/cybersecurity/dorothea/-/tree/master/labs/lab_attacks/attacks).

Differences between $D_{1}$ and $D_{2}$ were found in malicious flow data. On $D_{1}$, port-scanning attacks were continuously launched. On $D_{2}$, slow port-scanning attacks were carried out. Specifically, requests were launched with 5 to 10 s of slack time between them. Slack time makes the attacking duration exponentially grow.

Networks’ IP address spaces were different at $D_{1}$ and $D_{2}$. On $D_{1}$, the IP address space was 182.168.1.1/24 for benign-traffic simulators and attack nodes; the 126.52.30.0/24 address space was used for victim nodes. On $D_{2}$, the IP address space was 152.148.48.1/24 for benign-traffic simulators and attack nodes, while 140.30.20.1/24 address space was used for victim nodes.

### 3.2. Classification-Model Fitting

To evaluate DOROTHEA, several classification models were fitted with the above datasets. To train and test the classification models, the model evaluator (MoEv) tool was used. MoEv is a general-purpose tool for building classification models from labeled datasets (MoEv’s technical report is available online (10.5281/zenodo.4114127)). In addition, MoEv cleans data and performs other preprocessing operations. It was successfully used in different research areas such as jamming-attack detection in real-time location systems [21], or academic-success prediction at educational institutions [22]. However, it has not been validated on malicious-network-traffic detection yet. Therefore, we evaluated the classification models’ performance built with MoEv for malicious-traffic detection. In order to do so, we replicated the experiments carried out in [23].

In [23], the authors used the CICIDS2017 dataset to build classification models for cyberattack detection. The CICIDS2017 dataset contains network traffic that resembles true real-world data. It contains benign and malicious traffic from the most common attacks, including port scanning. It also gathers the results of network-traffic analysis using CICFlowMeter with labeled flows based on the timestamp, source, and destination IPs, source and destination ports, protocols, and attacks. Labeled flows for machine- and deep-learning purposes are publicly available to researchers [24]. This specific work was selected since its authors used flow data to obtain the classification models. We aimed to replicate their results and conclusions in order to empirically demonstrate that MoEv was able to build classification models from flow data.

On the experiments carried out in [23], the CICIDS2017 dataset was preprocessed. First, it was cleaned by removing redundant and non-significant features, replacing NaN values with zero values, and replacing infinite values with the mean value. Then, feature reduction was applied according to their variance. Any feature with zero variance was removed. Lastly, principal-component analysis was carried out to further reduce the number of features. In order to work with the same data, we performed the same preprocessing except for the last step. Instead, the most significant features were selected according to the K highest scores since this method was implemented in MoEv.

In [23], according to the obtained accuracy scores, classification models with a higher detection rate for port-scanning attacks were KNN, decision tree, and NB. The classification models’ accuracy was computed as shown in Equation (1), where $T_{P}$ is the true-positive rate, $T_{N}$ is the true-negative rate, $F_{P}$ is the false-positive rate, and $F_{N}$ is the false-negative rate.

(1) $$accuracy=\frac{T_{P}+T_{N}}{T_{P}+F_{P}+T_{N}+F_{N}}$$

To replicate the above study, we used MoEv to build classification models using KNN, decision-tree, and NB algorithms. Then, we compared the obtained results at [23] with ours. Since the accuracy scores of the classification models were similar, we could empirically assert that MoEv could build classification models for malicious-traffic detection from flow data.

Next, we aimed to build new classification models with MoEv from datasets gathered with DOROTHEA. We used data from $D_{1}$ to fit the models; 80% of flow data were used for training models and 20% to test them. MoEv evaluates classification, clustering, and regression algorithms to select the most accurate for a specific problem. The following algorithms were computed: Adaptive boosting (AB) [25], Bagging classifier (BC) [26], Bernoulli restricted Boltzmann machine (BRBM) [27], Classification and regression tree (CART) [28], KNN [29], Linear discriminant analysis (LDA) [30], Logistic regression (LR) [31], NB [32], One-vs-the-rest (OvR) [33], Quadratic discriminant analysis (QDA) [34], Random forest (RF) [35], Stochastic gradient descent (SGD) [36].

MoEv carries out k-iteration cross-validation to identify the most accurate classification algorithm. Due to selecting it, a set of well-known Key performance indicators (KPIs) were computed. First, model performance was measured by considering their accuracy score (see Equation (1)). In order to ensure model generalization, we tested the above models with data from both $D_{1}$ and $D_{2}$.

Lastly, models with the highest accuracy scores were preselected for in-depth evaluation by considering the following KPIs obtained through the confusion matrix: Precision (P), Recall (R), and $F_{1}$ score ($F_{1}$). P, R, and $F_{1}$ were computed as shown in Equations (2)–(4), respectively.

(2) $$P=\frac{T_{P}}{T_{P}+F_{P}}$$

(3) $$R=\frac{T_{P}}{T_{P}+F_{N}}$$

(4) $$F_{1}=2\frac{P\times R}{P+R}$$

A Jupyter notebook that allowed for replicating the evaluation is available online in a Binder-ready repository (https://niebla.unileon.es/cybersecurity/moev).

## 4. Results

Table 1 shows the accuracy-score comparison between the classification models proposed in [23] and the models built with MoEv. Accuracy obtained for the KNN and decision tree classifiers was the same, specifically, 99.9%. However, the accuracy of the naive Bayes classifier was significantly different, 98% versus 70%.

Regarding the two datasets gathered by using DOROTHEA, Table 2 shows the number of flows of legitimate and malicious traffic. At $D_{1}$, the malicious-flow percentage is 45.2. At $D_{2}$ the malicious-flow percentage is 48.3.

Table 3 shows the accuracy score of the classification models built with MoEv. Models were fitted carrying out 10 iterations of cross-validation by using data from $D_{1}$. The training accuracy score is shown in the second column. Next, in order to ensure optimal generalization, models were tested by using data from $D_{2}$. The accuracy test score is shown in the third column.

Models with a test score higher than 0.9 were in-depth analyzed by considering additional KPIs. Table 4 shows P, R, and $F_{1}$ scores for such models.

Results from Table 3 and Table 4 can be reviewed online through a Binder-ready repository that allows for replicating the evaluation (https://niebla.unileon.es/cybersecurity/moev).

## 5. Discussion

Results in Table 1 show that the accuracy score obtained with KNN and decision-tree algorithms was the same for the models proposed in [23] and models built with MoEv. The only difference was when the naive Bayes algorithm was used. With this algorithm, the obtained accuracy in [23] was higher than the accuracy obtained with MoEv. We attribute this difference to the usage of different hyperparameters in MoEv, since the authors in [23] did not provide the used parameters in this model. According to the above, we can empirically assert that MoEv allows for malicious traffic detection, since the experiments proposed in [23] were successfully replicated.

Regarding data gathering, Table 2 shows the number of flows in $D_{1}$ and $D_{2}$. In both datasets, the malicious-traffic rate was similar, 45.2% versus 48.3%. A high malicious-traffic rate is required in order to build classification models for malicious-traffic detection since most classification algorithms require enough data to find patterns; $D_{1}$ and $D_{2}$ gather flow data instead of network packets, like other popular datasets such as NSL-KDD and UNSW-NB15. Consequently, $D_{1}$ and $D_{2}$ are suitable to train malicious-traffic detection models by analyzing flow data.

Once we had demonstrated that MoEv was a valid tool to fit models for malicious-traffic detection, the next step was to use it for fitting models by using data from datasets gathered with DOROTHEA. Table 3 shows the accuracy scores by using $D_{1}$ and $D_{2}$ datasets. The accuracy scores by using $D_{1}$ were 100% in most cases. However, this was not enough to assert that the classification models were valid to detect port-scanning attacks. Moreover, we had to ensure their generalization by using a different dataset. Models were tested using $D_{2}$. Accuracy scores decreased in all cases by using $D_{2}$. However, there were 7 classifiers with an accuracy score higher than 0.9. These promising models were in-depth analyzed by considering additional KPIs.

Table 4 shows P, R, and $F_{1}$ scores for classification models with an accuracy score higher than 0.9 using $D_{2}$. The P score shows the ratio between the number of correct predictions (both positive and negative) and the total number of predictions. The R score shows the rate of positive cases that were correctly identified by the algorithm. The $F_{1}$ score relates both P and R since it was their harmonic mean. In order to detect all malicious flows, we required a high R score, but only if the P score was also high enough to ensure that there were not too many false negatives. Thus, the $F_{1}$ score was the most important KPI to consider. The KNN classifier had the highest P, R, and $F_{1}$ scores. So, after our analysis, KNN was the best classifier. KNN is also one of the most used algorithms in the literature to detect malicious traffic, as shown in [6]. In [6], the authors established that decision trees and NB are commonly used to detect malicious traffic. In our research, decision trees also presented good performance with accuracy, P, R, and $F_{1}$ scores higher than 0.9. Regarding NB, despite it presenting a high accuracy score using $D_{1}$, it did not ensure generalization since its accuracy considerably decreased when testing with $D_{2}$. We attribute this difference to most works described in [6] not including a validation step. Thus, we can empirically assert that models fitted with data gathered by DOROTHEA could detect malicious traffic.

## 6. Conclusions

Malicious-traffic detection is a powerful method to detect APTs. To detect such malicious traffic, classification models are the most used tools. However, to build such models, suitable datasets are required containing both legitimate and malicious network traffic. In addition, when detection must be done at routers with a high packet-per-second rate, pcap-based datasets are not suitable. Considering the above, this paper described a framework to gather packet-flow datasets for fitting classification models for malicious-traffic detection. We also presented DOROTHEA, a Docker-based solution.

In order to demonstrate that datasets gathered with DOROTHEA fit classification models for malicious-traffic detection, several classification models were built using MoEv. MoEv is a general-purpose tool for fitting classification models. It was successfully used to detect cyberattacks on location systems and for predicting academic success. In this work, it was also successfully used to build classification models to detect malicious traffic by using data from publicly available datasets, and replicating methods and experiments found in the literature.

Once MoEv was evaluated to build malicious-traffic detection models, it was used to create new models by using data gathered by DOROTHEA. The new models’ generalization was successfully evaluated by testing them with data from a different dataset. According to our research, the best algorithm to detect malicious traffic on flow data according to its accuracy score with a test dataset was KNN, which is also the most commonly used algorithm in the literature.

The most important contribution of this research is the proposed flow-data generation method. A Docker-based solution of the above proposal is presented, DOROTHEA. We empirically demonstrated that datasets gathered with DOROTHEA enables the building of malicious-traffic detection models. We used MoEv to build malicious-traffic detection models that ensured optimal generalization.

## Figures and Tables

**Table 1:** Accuracy comparison for k-nearest-neighbor (KNN), decision-tree, and naive Bayes classifiers.

Algorithm	Accuracy Obtained in [23]	Accuracy Using MoEv
KNN	99.9%	99.9%
Decision tree (1)	99.9%	99.9%
NB	98.0%	70.0%

(1) In [23], decision-tree algorithm was C4.5. MoEv used CART algorithm.

**Table 2:** Numbers of flows at $D_{1}$ and $D_{2}$.

Dataset	Legitimate Flows ${}^{\left(1\right)}$	Malicious Flows ${}^{\left(2\right)}$	Total Flows
$D_{1}$	1,429,038	1,178,992	2,608,030
$D_{2}$	600,767	560,209	1,160,976

${}^{\left(1\right)}$ Flow data labeled “0”. ${}^{\left(2\right)}$ Flow data labeled as “1”.

**Table 3:** Accuracy scores.

Classifier	Training Score ${}^{\left(1\right)}$	Test Score ${}^{\left(2\right)}$
KNN	100%	96.41%
LR	100%	94.83%
SGD	100%	92.10%
OvR	100%	93.23%
CART	100%	90.18%
RF	100%	90.18%
AB	100%	90.18%
BRBM	100%	78.22%
QDA	54.78%	51.71%
LDA	100%	51.71%
NB	100%	51.71%
BC	100%	51.71%

${}^{\left(1\right)}$ Accuracy score obtained after fitting models by using data from $D_{1}$. ${}^{\left(2\right)}$ Accuracy score obtained after testing models by using data from $D_{2}$.

**Table 4:** Accuracy, precision, recall, and $F_{1}$ score obtained by using data from $D_{2}$.

Classifier	Accuracy	Class ${}^{\left(1\right)}$	*P*	*R*	$F_{1}$ Score
KNN	0.964116	0	0.987845	0.942207	0.964486
		1	0.941017	0.987583	0.963738
		Avg.	0.965235	0.964116	0.964125
LR	0.948374	0	0.986233	0.912919	0.948161
		1	0.913607	0.986350	0.948586
		Avg.	0.951166	0.948374	0.948366
OvR	0.932330	0	0.985618	0.882021	0.930946
		1	0.886421	0.986215	0.933659
		Avg.	0.937722	0.932330	0.932256
SGD	0.920981	0	0.987506	0.858063	0.918245
		1	0.866689	0.988372	0.923540
		Avg.	0.929171	0.920981	0.920802
CART	0.901849	0	0.985478	0.822330	0.896542
		1	0.838361	0.987021	0.906637
		Avg.	0.914445	0.901849	0.901417
RF	0.901849	0	0.985478	0.822330	0.896542
		1	0.838361	0.987021	0.906637
		Avg.	0.914445	0.901849	0.901417
AB	0.901849	0	0.985478	0.822330	0.896542
		1	0.838361	0.987021	0.906637
		Avg.	0.914445	0.901849	0.901417

${}^{\left(1\right)}$ Benign flow data, “0”; malicious flow data, “1”.

## Abbreviations

The following abbreviations are used in this manuscript:

AB	Adaptive boosting
AODE	Averaged one-dependence estimator
APT	Advanced persistent threat
BC	Bagging classifier
BRBM	Bernoulli restricted Boltzmann machine
CART	Classification and regression tree
DOROTHEA	Docker-based framework for gathering netflow data
KNN	K-nearest neighbors
KPI	Key performance indicator
LDA	Linear discriminant analysis
MLP	Multilayer perceptron
MoEv	model evaluator
pcap	packet capture
NB	Naive Bayes
OvR	One-vs-the-rest
QDA	Quadratic discriminant analysis
RF	Random forest
SCAYLE	Supercomputación Castilla y León
SGD	Stochastic gradient descent
